# Cross-border investigation of a tuberculosis outbreak in Vienna linked to a multi-country cluster among foreign-born individuals, Europe, 2021 to 2025

**DOI:** 10.2807/1560-7917.ES.2025.30.33.2500565

**Published:** 2025-08-21

**Authors:** Fiona Költringer, Maria Koreny, Dirk Werber, Florian Heger, Alena Chalupka, Sabine Schweiger, Georg Brunner, Ursula Tuch, Ursula Karnthaler, Senia Rosales Klintz, Csaba Ködmön, Andreas Hoefer, Richard Anthony, Stefan Kroeger, Teresa Domaszewska, Lena Boes, Stefan Niemann, Teresa Walz, Martin Kuhns, Sarah Jackson, Margaret Fitzgibbon, Vanessa Mathys, Vinciane Sizaire, Daniela Maria Cirillo, Monica Sane Schepisi, Janne Oseberg Rønning, Laura Herrera-Leon, Pere-Joan Cardona, Ramona Groenheit, Mikael Mansjö, Viktória Szél, Erik Alm, Adriana Cabal

**Affiliations:** 1 Institute for Infectious Disease Epidemiology, Austrian Agency for Health and Food Safety, Vienna, Austria; 2 Tuberculosis Prevention and Control, Public Health Services, City of Vienna, Austria; 3 National Reference Centre for Tuberculosis, Institute for Medical Microbiology and Hygiene, Austrian Agency for Health and Food Safety, Vienna, Austria; 4 European Centre for Disease Prevention and Control (ECDC), Stockholm, Sweden; 5 Centre for Infectious Disease Control, National Institute for Public Health and the Environment (RIVM), Bilthoven, The Netherlands; 6 Infectious Disease Epidemiology Department, Robert Koch Institute, Berlin, Germany; 7 Research Center Borstel, National Reference Center for Mycobacteria, and Molecular and Experimental Mycobacteriology, Borstel, Germany; 8 Health Protection Surveillance Centre, Health Service Executive, Dublin, Ireland; 9 Irish Mycobacteria Reference Laboratory, St James’s Hospital, Dublin, Ireland; 10 National Reference Centre Mycobacterium, Bacterial Diseases Service, Sciensano, Brussels, Belgium; 11 Fonds des Affections Respiratoires (FARES), Brussels, Belgium; 12 Reference Laboratory for Tuberculosis Molecular Surveillance, IRCCS San Raffaele Scientific Institute, Milan, Italy; 13 Italian Ministry of Health, General Directorate for Health Prevention, Rome, Italy; 14 Norwegian Institute of Public Health, Department of Bacteriology, National Reference Laboratory for Mycobacteria, Oslo, Norway; 15 National Reference Laboratory for Mycobacteriology, National Microbiology Centre - Carlos III Institute of Health, Madrid, Spain; 16 Servei de Microbiologia, Laboratori Clínic Metropolitana Nord. Hospital Germans Trias i Pujol, Badalona, Catalonia, Spain; 17 Public Health Agency of Sweden, Stockholm, Sweden; 18 National Reference Laboratory for Mycobacteriology, Korányi National Institute for Tuberculosis and Respiratory Medicine, Budapest, Hungary

**Keywords:** Tuberculosis, Whole Genome Sequencing, Cluster, Cross-border, Genomic surveillance

## Abstract

Collaborative genomic and epidemiological investigations identified a tuberculosis outbreak in Vienna as part of a multi-country cluster comprising 57 foreign-born cases of *Mycobacterium tuberculosis* ST215/Beijing 2.2.1 notified 2021–2025. While 14 of 16 cases in Vienna were considered autochthonous, the diverse geographic origin of clustered cases across nine European countries suggests a common transmission source, possibly linked to migratory routes. Cross-border data exchange and integrated genomic analysis are essential for identifying transmission dynamics in tuberculosis clusters affecting mobile populations.

We present preliminary findings from an investigation of a newly identified *Mycobacterium tuberculosis* strain of sequence type (ST)215, lineage Beijing/2.2.1 associated with a cross-border cluster comprising 57 tuberculosis (TB) cases in nine European countries, first detected in Vienna, Austria. To assess its geographical extent, a collaborative epidemiological and genomic investigation was initiated.

## Outbreak detection and investigations in Austria

An increase in notified cases with Somali origin from 9% (n = 11) to 17% (n = 25) among all notified TB cases between 2022 and 2024 in Vienna, raised concerns about autochthonous transmission. Local public health authorities interviewed the patients and traced the contacts.

To identify clusters, we performed whole genome sequencing (WGS). Single-linkage core genome multilocus sequence typing (cgMLST) analysis with a cluster threshold of ≤ 12 alleles was conducted using Ridom Seqsphere + [1]. Firstly, the analysis included isolates from all TB cases with Somali origin notified by public health authorities in Vienna in 2023–2024 (n = 47). We identified one cluster comprising 14 cases with *M. tuberculosis* ST215-Beijing/2.2.1 with 0–1 allelic differences, along with two smaller clusters.

Secondly, isolates of the *M. tuberculosis* ST215-Beijing/2.2.1 cluster were compared against the sequencing database of the National Reference Center for Tuberculosis in Vienna, including 6,197 isolates from TB cases in Austria since 2015. Six additional cases were identified, clustering with the 14 previously identified cases within 0–4 allelic differences. These six cases were notified by five other Austrian federal states in individuals from Afghanistan (n = 2), Somalia, Morocco, Yemen and Pakistan. Thirdly, all isolates from new TB cases notified in 2025 (by 31 May) were added to the analysis, identifying two additional cases in Vienna and one case from outside Vienna, all in Somali-born individuals.

## Outbreak case definition and classification

We defined an outbreak case as a person with TB, notified in Austria between 1 January 2015 and 31 May 2025, with an isolate clustering within ≤ 12 allelic differences to the outbreak strain described above. Overall, we identified 23 outbreak cases notified between 2021 and 2025 in foreign-born individuals. Twenty of these 23 cases were screened for active pulmonary TB upon entry, and three cases presented cavernous lesions in chest X-rays. Patient characteristics are summarised in Table.

**Table:** Characteristics of patients in an outbreak of *Mycobacterium tuberculosis* sequence type (ST)215-Beijing/2.2.1, Austria, 2021–31 May 2025 (n = 23)

Characteristics	n
Sex	
Female	0
Male	23
Age (years)	
Median	25
IQR	21–28
Microscopy results	
Smear positive	6
Smear negative	14
Unknown	3
Disease presentation	
Pulmonary	16
Extrapulmonary	6
Pulmonary and extrapulmonary	1
Phenotypic antimicrobial susceptibility testing	
Susceptible to all first line antimicrobials	23
Method of case finding	
Chest X-ray screening at entry^a^	3
Diagnosed after clinical symptoms^b^	18
Accidental finding^c^	2
Completion of treatment^d^	
Lost to follow-up at 12 months^e^	3
Completed at 12 months	12
Still on treatment^f^	8
Country of birth	
Somalia	18
Afghanistan	2
Pakistan	1
Yemen	1
Morocco	1
Migration history	
Western Balkans route	10
Unknown	13

IQR: interquartile range; TB: tuberculosis.

^a^ Screening for active pulmonary tuberculosis with chest X-ray upon arrival in Austria.

^b^ Epidemiological links were confirmed for two cases.

^c^ TB diagnosis when investigating an unrelated medical issue.

^d^ Treatment according to the international guidelines [12].

^e^ Patient relocated to unknown residence before completing the full course of treatment.

^f^ As of 31 May 2025, treatment is ongoing.

Cases were classified as autochthonous if notified > 5 years after date of entry (n = 11) or > 2 years after date of entry and with negative screening results for active pulmonary TB upon entry (n = 4). These cutoffs were chosen based on the evidence that the risk of progression to active TB peaks within 2 years after infection and declines markedly by 5 years post infection [2-5]. To address the limitation that time since entry may not sufficiently account for late progression, entry screening results were incorporated into the classification criteria for an autochthonous case. Imported cases were defined as cases detected through entry screening (n = 3) or with reporting date < 6 months after date of entry (n = 1). This approach was chosen to ensure that only cases with strong evidence of pre-existing TB were included. Acknowledging the variable progression timelines of TB and its heterogeneous clinical presentations [6], the remaining cases were classified as undetermined (n = 4).

## Epidemiological findings

**Figure 1:**
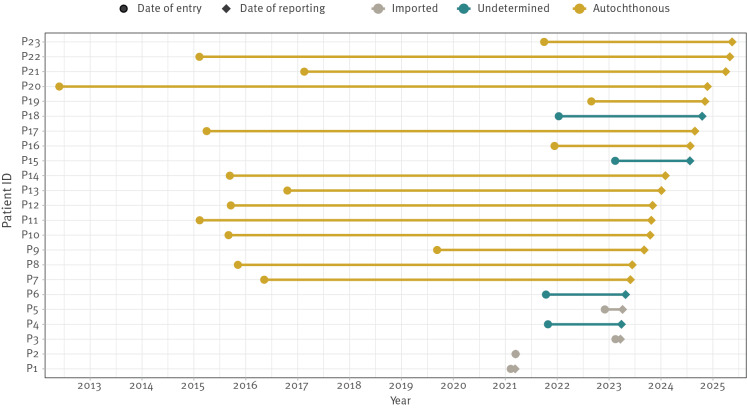
Classification of cases as autochthonous or imported in an outbreak of *Mycobacterium tuberculosis* sequence type (ST)215-Beijing/2.2.1, Austria, 2015–2025 (n = 23)^a,b^

^a^ Cases were classified based on the time elapsed between their date of entry and date of reporting.

^b^ Patient 4 (P4) was identified as the suspected primary case of the outbreak in Vienna.

The median time between date of entry and notification was 4.1 years (interquartile range (IQR): 1.5–8.3). Among the 15 cases classified as autochthonous, epidemiological links could be confirmed for two individuals (P8 and P19, Figure 1). All cases classified as autochthonous were born in Somalia. Of the 16 cases notified by Vienna, 14 were classified as autochthonous. Three imported cases were identified through entry screening and correspond to the earliest cases involving individuals from Pakistan, Afghanistan and Morocco. Two undetermined cases in Vienna reported arrival to Austria along the Western Balkans route.

In Vienna, epidemiological investigations identified a suspected primary case (P4, Figure 1) who migrated from Somalia to Austria via the Western Balkans route arriving in October 2021. Formally classified as undetermined, the case was reported with TB 17 months after arrival, aligning with the onset of local transmission in Vienna. The chest X-ray on hospital admission displayed cavernous lesions, suggesting that the patient had been infectious during the previous 6 months while still socially active.

## EpiPulse alert and collaborative cluster investigation

Due to concerns regarding potential cross-border transmission, Austria issued an alert on the outbreak via EpiPulse in February 2025 [7]. Countries were asked to screen their databases for matching isolates using a representative outbreak strain provided by Austria. Of the 13 responding countries, 10 countries, including Austria, submitted short-read data to European Centre for Disease Prevention and Control (ECDC). Single nucleotide polymorphisms (SNP) analysis was performed using Snippy version 4.6.0 (https://software.pureos.net/package/src/pureos/landing/snippy) with default parameters and H37Rv v3 as genome reference available in GenBank (https://www.ncbi.nlm.nih.gov/nuccore/448814763). A maximum likelihood tree was built using annotated epidemiological data provided by the submitting countries. In this report, the term outbreak refers to the Austrian event, whereas the cross-border occurrence is referred to as a cluster, given that epidemiological links between cases have yet to be confirmed.

**Figure 2:**
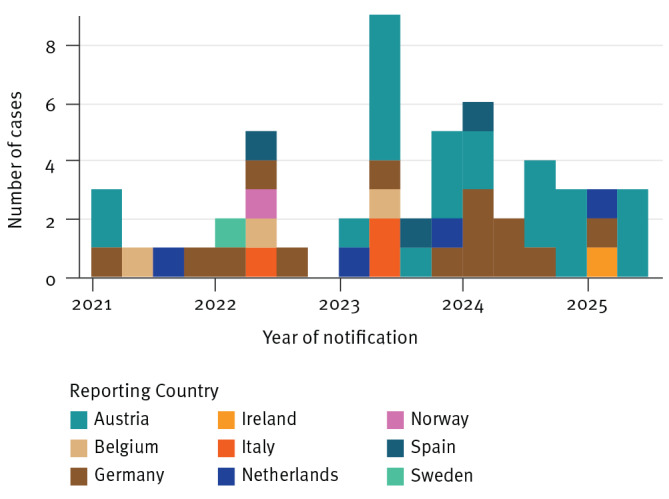
Clustered cases with *Mycobacterium tuberculosis* sequence type (ST)215-Beijing/2.2.1, by notification year of and country of origin, Austria, Belgium, Germany, Ireland, Italy, the Netherlands, Norway, Spain and Sweden, March 2021–April 2025 (n = 53)^a,b^

^a^ Four German cases are not displayed due to missing notification dates.

^b^ If the notification date was not available, the date of diagnosis or the date of first positive sampling was used.

**Figure 3:**
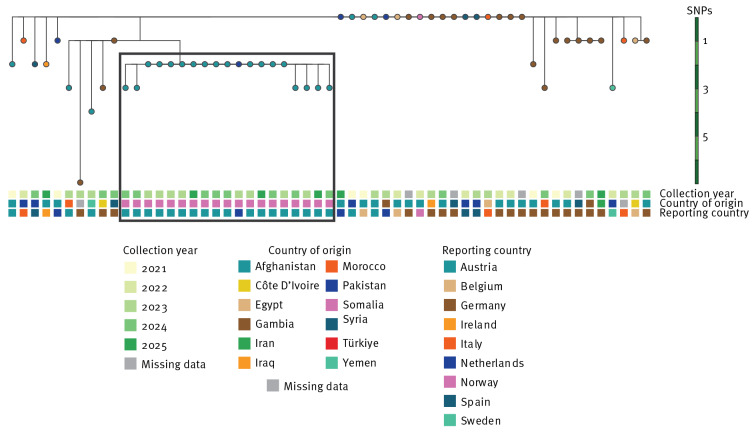
Maximum likelihood tree of *Mycobacterium tuberculosis* sequence type (ST)215-Beijing/2.2.1 isolates matching the cluster case definition, EU/EEA, 2021 to 2025 (n = 57)^a^

ECDC: European Centre for Disease Prevention and Control; EU/EEA: the European Union and European Economic Area; SNP: single nucleotide polymorphism.

^a^ The tree was built using Snippy version 4.6.0 with default parameters (minimum local coverage 10 × and minimum variant quality 100) and using H37Rv as the reference genome. Two closely related isolates from the ECDC sequence collection, around 30 SNPs distant from the cluster (not shown), were included in the SNP analysis and used together with the reference genome as outgroups to root the tree. The tree nodes are coloured according to reporting country and the annotation blocks are coloured according to sample collection year, country of origin and reporting country. The sub-cluster linked to patients with Somalian origin is highlighted by a rectangle.

Tuberculosis cases notified in the European Union and European Economic Area (EU/EEA) countries before 31 May 2025 with an isolate of *M. tuberculosis* ST215–Beijing/2.2.1 with ≤ 10 SNPs compared with the representative strain were considered part of the cluster. A total of 57 cases notified between 2021 and 2025 were identified in nine EU/EEA countries (Figure 2). All isolates (n = 19) from cases born in Somalia were captured in a sub-branch of the phylogenetic tree, including 18 from Austria and one from the Netherlands (Figure 3). The remaining isolates, which were more ancestral than the reference genome, were predominantly from individuals originating from Afghanistan (n = 14) and 10 other countries. Details on the SNPs characteristics of the clusters are presented in Supplementary Table S1. No isolates were reported as phenotypically resistant nor presented mutations conferring antimicrobial resistance to first-line TB drugs including rifampicin, isoniazid, ethambutol and pyrazinamide.

## Discussion

Preliminary findings suggest that the TB outbreak among Somali-born individuals in Vienna is associated with an ongoing cross-border cluster affecting foreign-born individuals across nine EU/EEA countries. Several factors indicate that autochthonous transmission is likely contributing to the outbreak in Austria. First, most cases were notified years after resettlement. Second, despite a high TB burden in Somalia [8], low prevalence of the identified outbreak strain in Somalia [9,10] suggests that direct importation of infections is unlikely. Third, 18 of 23 cases in Austria were born in Somalia, whereas only one case of Somali origin was notified by other countries.

In contrast to Austria, detections in other countries predominantly involved individuals of Afghan origin. The earliest cases of the cluster were traced to Afghan individuals (n = 5) indicating a possible origin of the ancestral strain. Most other clustered cases also originated from high TB burden countries [8]. However, the narrow clustering (0–3 SNPs) among cases from diverse geographic origins across multiple EU/EEA countries points to a common transmission source rather than to multiple independent introductions. Such common transmission sources may be linked to migratory routes, as previously observed in a TB cluster among individuals migrating from the Horn of Africa to the EU/EEA region [11].

Plausible migration routes of the cases identified in other affected countries do not transit through Austria, supporting the assumption that the strain was introduced to Vienna rather than originating there. Although details on timing and location of exposure remain unavailable, and transmission following arrival in Austria cannot be excluded, the near-complete sequencing coverage of the isolates from the TB cases within the national surveillance system suggests that the primary case in Vienna likely acquired the infection during their migration journey. Additional epidemiological investigations on migration histories are needed to identify a potential common source of transmission.

## Conclusion

This multi-country TB cluster likely emerged from a combination of transmission dynamics, including introduction from countries of origin, infection through a common source, possibly along an established migratory route, and autochthonous transmission within destination countries. The rapid identification of related isolates was made possible through cross-border genomic data exchange facilitated by EpiPulse. Integrated genomic and epidemiological investigations are essential in understanding transmission patterns within TB clusters to identify settings where targeted interventions could most effectively interrupt transmission pathways.

## Data Availability

Whole genome sequencing (WGS) data for the Austrian isolates are available under PRJEB96240.

## Supplementary Data

32000 Supplementary Material
