# Author’s correction for Euro Surveill. 2025;30(33)

**DOI:** 10.2807/1560-7917.ES.2025.30.42.250925c

**Published:** 2025-10-23

**Authors:** Eurosurveillance editorial team

**Affiliations:** 1 European Centre for Disease Prevention and Control (ECDC), Stockholm, Sweden

In the article ‘Cross-border investigation of a tuberculosis outbreak in Vienna linked to a multi-country cluster among foreign-born individuals, Europe, 2021 to 2025’ by Költringer et al., published on 21 August 2025, Laura Gavaldà-Mestre was missing from the list of authors at the time of publication. All listed authors have confirmed their consent to add Laura Gavaldà-Mestre as a co-author and the name was added on 21 October 2025 at the request of the authors.

